# HIV care cascade for women living with HIV in the Greater Toronto Area versus the rest of Ontario and Canada

**DOI:** 10.1177/09564624221108034

**Published:** 2022-11-21

**Authors:** Priscilla Medeiros, Laura Warren, Mina Kazemi, Notisha Massaquoi, Stephanie Smith, Wangari Tharao, Lena Serghides, Carmen H Logie, Abigail Kroch, Ann N Burchell, Alexandra de Pokomandy, Angela Kaida, Mona Loutfy, On behalf of the CHIWOS Research Team

**Affiliations:** 1 Women’s College Research Institute, 7985 Women’s College Hospital, Toronto, ON, Canada; 2 Dalla Lana School of Public Health, 7938 University of Toronto, Toronto, ON, Canada; 3 Faculty of Social Work, 7938 University of Toronto, Toronto, ON, Canada; 4 Women’s Health in Women’s Hands Community Health, Toronto, ON, Canada; 5 Toronto General Hospital Research Institute, 7989 University Health Network, Toronto, ON, Canada; 6 Department of Immunology, 7938 University of Toronto, Toronto, ON, Canada; 7 269770 Ontario HIV Treatment Network, Toronto, ON, Canada; 8 Department of Family and Community Medicine, 548628 St. Michael’s Hospital, Toronto, ON, Canada; 9 Faculty of Medicine, 7938 University of Toronto, Toronto, ON, Canada; 10 Chronic Viral Illness Service, 507266 McGill University Health Centre, Montreal, QC, Canada; 11 Department of Family Medicine, McGill University, Montreal, QC, Canada; 12 Faculty of Health Sciences, 1763 Simon Fraser University, Burnaby, BC, Canada; 13 Faculty of Medicine, 7938 University of Toronto, Toronto, ON, Canada

**Keywords:** Women, Greater Toronto Area, HIV cascade of care, Canada, CHIWOS

## Abstract

**Background:**

The Greater Toronto Area (GTA) is home to 39% of Canada’s population living with HIV. To identify gaps in access and engagement in care and treatment, we assessed the care cascade of women living with HIV (WLWH) in the GTA versus the rest of Ontario and Canada (in this case: Quebec and British Columbia).

**Methods:**

We analyzed 2013–2015 self-reported baseline data from the Canadian HIV Women’s Sexual and Reproductive Health Cohort Study for six care cascade stages: linked to care, retained in care, initiated antiretroviral therapy (ART), currently on ART, ART adherence (≥90%), and undetectable (<50 copies/mL). Multivariable logistic regression was used to reveal associations with being undetectable.

**Results:**

Comparing the GTA to the rest of Ontario and Canada, respectively: 96%, 98%, 100% were linked to care; 92%, 94%, 98% retained in care; 72%, 89%, 96% initiated ART; 67%, 81%, 90% were currently using ART; 53%, 66%, 77% were adherent; 59%, 69%, 81% were undetectable. Factors associated with viral suppression in the multivariable model included: living outside of the GTA (Ontario: aOR = 1.72, 95% CI: 1.09–2.72; Canada: aOR = 2.42, 95% CI: 1.62–3.62), non-Canadian citizenship (landed immigrant/permanent resident: aOR = 3.23, 95% CI: 1.66–6.26; refugee/protected person/other status: aOR = 4.77, 95% CI: 1.96–11.64), completed high school (aOR = 1.77, 95% CI: 1.15–2.73), stable housing (aOR = 2.13, 95% CI: 1.33–3.39), income of ≥$20,000 (aOR = 1.52, 95% CI: 1.00–2.31), HIV diagnosis <6 years (6–14 years: aOR = 1.75, 95% CI: 1.16–2.63; >14 years: aOR = 1.87, 95% CI: 1.19–2.96), and higher resilience (aOR = 1.02, 95% CI: 1.00–1.04).

**Conclusion:**

WLWH living in the GTA had lower rates of viral suppression compared to the rest of Ontario and Canada even after adjustment of age, ethnicity, and HIV diagnosis duration. High-impact programming for WLWH in the GTA to improve HIV outcomes are greatly needed.

## Introduction

The Paris Declaration was launched on World AIDS Day 2014. Since then, more than 300 cities and municipalities have committed to ending the AIDS epidemic by 2030.^1,2^ Many of these cities are known as Fast-Track Cities, aiming to meet the Joint United Nations Programme on HIV/AIDS 90–90-90 targets: 90% of people living with HIV (PLWH) will know their HIV status, 90% of those diagnosed will receive receiving antiretroviral therapy (ART), and 90% of those receiving ART will be virally suppressed.^3^ Cities play a critical role in fast tracking the response to HIV and ending the AIDS epidemic by 2030, as many PLWH move to these larger cities to access care.^4^

Toronto, also known as the Greater Toronto Area (GTA), is the largest city in Canada and is one of the cities that has signed on to be a Fast Track City.^5^ Known as “Toronto to Zero,” the aim is to reduce the number of new HIV transmissions in the city and surrounding Greater Toronto Area (GTA) annually, to ensure that populations most affected by HIV have access to care, rapidly begin taking ART, and rapidly achieve viral suppression, to improve quality of life for all PLWH, and to end HIV stigma and discrimination.^5^

There are 75,500 PLWH in all of Canada with 35,122 living in Ontario and 16,228 living in the GTA, nearly half and one-fifth, respectively. Of the reported HIV cases in Canada, Ontario, and the GTA, 16,880, 8,000, and 4,057 are women, respectively.^5–8^ The two main groups of women affected by HIV in the GTA and Ontario are African, Caribbean, and Black (ACB) women, many of whom have immigrated to Canada, and women who inject drugs.^7^ An estimated 46% of all women living with HIV (WLWH) in the GTA reported emigrating to Canada from a country with high HIV prevalence.^5^

Women living with HIV have lower engagement and retention across the HIV care cascade compared to men living with HIV in North America.^9–11^ In Ontario, an estimated 80% of men living with HIV are virally suppressed compared to 77% of WLWH.^12^ To date, there has not been an assessment of HIV care cascade indices by gender in the GTA, which is necessary to inform the Toronto to Zero endeavor.

Using data collected from the Canadian HIV Women’s Sexual and Reproductive Health Cohort Study (CHIWOS), the aims of our study were to determine: (1) the sociodemographic and clinical differences between WLWH in the GTA versus in the rest of Ontario and the rest of Canada, Quebec and British Columbia [BC]; (2) the differences in the HIV care cascade stages between WLWH in the GTA versus in the rest of Ontario, Quebec and BC; and (3) to compare which characteristics predict viral suppression among WLWH in the entire CHIWOS cohort and those living in the GTA.

## Methods

### Study design

The current study used baseline peer research associates-administered survey data from CHIWOS, a national community-based cohort study examining the health and healthcare priorities of WLWH from the provinces of BC, Ontario, and Quebec over the past decade. In CHIWOS, 1422 WLWH were enrolled from August 2013 to May 2015. More detailed descriptions of the study have been previously published.^13–16^

### Covariates

The main explanatory variable of interest was area of residence: GTA versus the rest of Ontario (i.e. excluding the GTA) and versus the rest of Canada (Quebec and BC).

Covariates of interest (shown in Table 1) included: age (continuous and categorical); sexual orientation; legal relationship status; immigration status; ethnicity; education; housing stability; personal annual income in Canadian dollar (CAD); food security; any violence as a child; cannabis use; history of incarceration; duration of HIV diagnosis; and hepatitis C infection

**Table 1.** Baseline sociodemographic and clinical characteristics of women living with HIV in the Greater Toronto Area (GTA) versus the rest of Ontario, and in Quebec and British Columbia (*n* = 1,422).

Variables	GTA (*n* = 388)/n (%)*	Rest of ON (*n* = 326)/n (%)*	QC and BC (*n* = 708)/n (%)*	*p*-value
Sociodemographic factors				
Age (year), median (IQR) (*n* = 1,422)	38.0 (33.0, 46.0)	42.0 (36.0, 51.0)	45.0 (37.0, 52.0)	<0.0001
Sexual orientation (*n* = 1,422)				
Heterosexual	329 (84.8)	289 (88.7)	619 (87.4)	0.48
LGBTTQ	55 (14.2)	37 (11.4)	88 (12.4)	
DK/PTNA†	4 (1.0)	0	1 (0.1)	
Legal relationship status (*n* = 1,422)				
Married/common-law/in relationship	113 (29.1)	109 (33.4)	232 (32.8)	<0.0001
Single	230 (59.3)	149 (45.7)	310 (43.8)	
Separated, divorced, widowed, other	44 (11.3)	68 (20.9)	165 (23.3)	
DK/PTNA†	1 (0.3)	0	1 (0.1)	
Immigration status (*n* = 1,422)				
Canadian citizen	292 (75.3)	272 (83.4)	589 (83.2)	<0.01
Landed immigrant/permanent resident	50 (12.9)	41 (12.6)	76 (10.7)	
Refugee/protected person	31 (8.0)	6 (1.8)	26 (3.7)	
Refugee claimant/person in need of protection	13 (3.4)	5 (1.5)	16 (2.3)	
Undocumented immigrant	2 (0.5)	2 (0.6)	1 (0.1)	
Ethnicity (*n* = 1,419)				
Indigenous	87 (22.5)	62 (19.1)	169 (23.9)	<0.0001
African/Caribbean/Black	146 (37.8)	86 (26.5)	194 (27.4)	
White	121 (31.4)	163 (50.2)	311 (43.9)	
Other ethnicity	32 (8.3)	14 (4.3)	34 (4.8)	
Education (*n* = 1,422)				
Lower than high school	33 (8.5)	48 (14.7)	146 (20.6)	<0.0001
High school or higher	354 (91.2)	276 (84.7)	558 (78.8)	
DK/PTNA†	1 (0.3)	2 (0.6)	4 (0.6)	
Housing status (*n* = 1,422)				
Stable	327 (84.3)	302 (92.6)	641 (90.5)	<0.001
Unstable	61 (15.7)	24 (7.4)	67 (9.5)	
Personal annual income(CAD) (*n* = 1,422)				
<$20,000	237 (61.1)	227 (69.6)	534 (75.4)	<0.0001
$20,000–$40,000	78 (20.1)	60 (18.4)	106 (15.0)	
≥$40,000	60 (15.5)	32 (9.8)	53 (7.5)	
DK/PTNA†	13 (3.4)	7 (2.2)	15 (2.1)	
Food security (*n* = 1,416)				
Food insecure	278 (71.8)	201 (62.0)	428 (60.7)	<0.001
Food secure	109 (28.2)	123 (38.0)	277 (39.3)	
History of violence as child (*n* = 1,422)				
Yes	235 (60.6)	182 (55.8)	480 (67.8)	<0.001
No	127 (32.7)	111 (34.1)	171 (24.2)	
DK/PTNA†	26 (6.7)	33 (10.1)	57 (8.1)	
Psychosocial factors				
Cannabis use (*n* = 1,421)				
Yes (regularly/occasionally)	69 (17.8)	109 (33.4)	184 (26.0)	<0.0001
Former	32 (8.3)	69 (21.2)	171 (24.2)	
Never	276 (71.1)	146 (44.8)	342 (48.4)	
DK/PTNA†	11 (2.8)	2 (0.6)	10 (1.4)	
History of incarceration (*n* = 1,422)				
Never	306 (78.9)	202 (62.0)	388 (54.8)	<0.0001
Ever, but not last year	69 (17.8)	100 (30.7)	262 (37.0)	
Last year	12 (3.1)	24 (7.4)	56 (7.9)	
DK/PNTA	1 (0.3)	0 (0.0)	2 (0.3)	
Probable depression (CES-D), mean (SD) (*n* = 1,422)	8.5 (7.7)	10.1 (7.3)	10.8 (7.5)	<0.0001
Clinical factors				
Duration of HIV diagnosis(year) (*n* = 1,422)				
<6	143 (36.9)	70 (21.5)	132 (18.6)	<0.0001
6–14	144 (37.1)	132 (40.1)	276 (39.0)	
>14	85 (22.0)	115 (35.3)	277 (39.1)	
DK/PTNA†	16 (4.0)	9 (2.8)	23 (3.3)	
Stigma and discrimination				
HIV-related stigma (HSS), mean (SD) (*n* = 1,401)	62.1 (18.5)	57.1 (21.1)	54.5 (19.8)	<0.0001
Everyday discrimination racism, mean (SD) (*n* = 1,422)	21.9 (10.5)	17.7 (11.9)	18.1 (10.7)	<0.0001
Everyday discrimination sexism, mean (SD) (*n* = 1,422)	21.8 (10.3)	19.0 (10.3)	18.6 (9.7)	<0.0001
Well-being				
Resilience, mean (SD) (*n* = 1,422)	63.2 (7.4)	61.3 (8.2)	62.1 (8.3)	<0.01
Social support (MOS-SSS), mean (SD) (*n* = 1,422)	14.8 (4.4)	14.2 (4.7)	13.8 (4.3)	<0.01
Physical HRQOL, mean (SD) (*n* = 1,422)	47.29 (12.1)	43.93 (13.6)	42.45 (15.4)	<0.001
Mental HRQOL, mean (SD) (*n* = 1,422)	45.30 (14.5)	40.28 (14.1)	40.51 (13.9)	<0.001

Note: Categorical variables summarized as frequencies (n) and proportions (%) and continuous variables summarized as medians with interquartile ranges or means with standard deviations and specified by GTA: Greater Toronto Area; ON: Ontario; QC: Quebec; BC: British Columbia; LGBTTQ: Lesbian, Gay, Bisexual, Transgender, Two-Spirit, and Queer or Questioning; DK/PTNA†: do not know/prefer not to answer; HSS: HIV Stigma Scale; SD: standard deviation; CES-D: Center for Epidemiological Studies Depression Scale; MOS-SSS: Social Support Scale; HRQOL: health-related quality of life.

Scales of interest included: HIV-related stigma (10-item HIV Stigma Scale; HSS);^17^ experiences of racism [Everyday Discrimination Racism scale (8-item scale)];^18^ experiences of gender discrimination [(Everyday Discrimination Sexism scale (8-item scale)];^18^ probable depression [10-item Center for Epidemiologic Studies Depression scale (CES-D)]^19,20^ resilience [10-item version of the resilience scale (RS-10)^21^]; social support [4-item Medical Outcomes Study Social Support scale (MOS-SSS)],^22,23^ and; physical health-related quality of life, and mental health-related quality of life score using the SF-12 scale.^24^

### Data analyses

Standard descriptive statistics were used to summarize the sociodemographic and clinical characteristics for WLWH in the GTA, rest of Ontario, and in Quebec and BC. Univariate associations were assessed using Chi-square testing for categorical variables and Student t-test or Wilcoxon rank-sum test for continuous variables when appropriate.

We calculated frequencies (n) and percentages (%) for each stage of the HIV care cascade stratified by women living in the GTA, rest of Ontario, and Quebec and BC.

The six stages of the HIV care cascade were: (1) linked to care; (2) retained in HIV care; (3) ART initiation; (4) current ART use; (5) ART adherence; and (6) viral suppression, and comparisons were made between regions for each stage using the Chi-square test.^21,25,26^ To appreciate the degree of attrition between stages, the percentage change for each stage was calculated using the number of “yes” responses from the previous stage minus the number of “yes” and “don’t know/prefer not to answer” responses in the current stage divided by the number of “yes” responses in the previous stage minus the number of “don’t know/prefer not to answer” responses in the current stage.^27^

Univariate and multivariable logistic regression analyses were conducted to determine the associations between the three geographic regions and other covariates with viral suppression. A *p*-value ≤ 0.20 was used for initial inclusion of covariates in the multivariable model for the entire CHIWOS cohort. All scales were included in the initial multivariable model irrespective of statistical significance. The same initial multivariable model developed for the entire CHIWOS cohort was used for the GTA cohort. A hierarchical manual backward stepwise elimination process was used to remove covariates from the multivariable models whose *p*-values exceeded 0.05. All statistical analyses were performed using SAS^®^ software version 9.4 (SAS Institute Inc., Cary, NC, US).

## Results

### Baseline characteristics of WLWH in the GTA versus in the rest of Ontario and in Quebec and BC

Of the 1422 WLWH in CHIWOS, 388 (27%) were from the GTA, 326 (23%) from the rest of Ontario, and 708 (50%) from Quebec and BC (Table 1). We found that WLWH in the GTA were younger with a median age of 38 [interquartile range (IQR), 33–46] versus 42 (IQR, 36–51) for those in the rest of Ontario and 45 (IQR, 37–52) in Quebec and BC (*p* =< 0.0001). Also, WLWH in the GTA were more gender diverse, although the difference was not statistically significant, with 14% identifying as LGBTTQ versus 11% in the rest of Ontario and 12% in Quebec and BC (*p* = 0.48).

Of the 388 women in the GTA, 38% identified as ACB, 23% as Indigenous, 31% as white, and 8% reported other ethnicities. Around half of the women in the rest of Ontario (50%) and in Quebec and BC (44%) identified as white, and the rest identified as ACB (ON 27% vs QC/BC 27%), Indigenous (ON 19% vs QC/BC 24%), and other ethnicities (ON 4% vs QC/BC 5%; *p* = 0.0001). Ninety-one percent of the women in the GTA reported completing high school or higher education, yet 61% of women in the GTA earned less than $20,000 per year. There were similar findings in the rest of Ontario and in Quebec and BC where women reported higher levels of education (ON 85% vs QC/BC 79%), and low-income earnings less than $20,000 per year (ON 70% vs QC/BC 75%). Women living in the GTA had also been diagnosed with HIV more recently, with 37% living with HIV for 6 years or less as compared to 22% of women in the rest of Ontario and 19% in Quebec and BC. Housing instability was more common (16%) among women living in the GTA compared to the rest of Ontario (7%) and in Quebec and BC (10%) (*p* < 0.01), as was food insecurity.

### HIV care cascade for WLWH in the GTA versus in the rest of Ontario and in Quebec and BC

**Figure 1.**
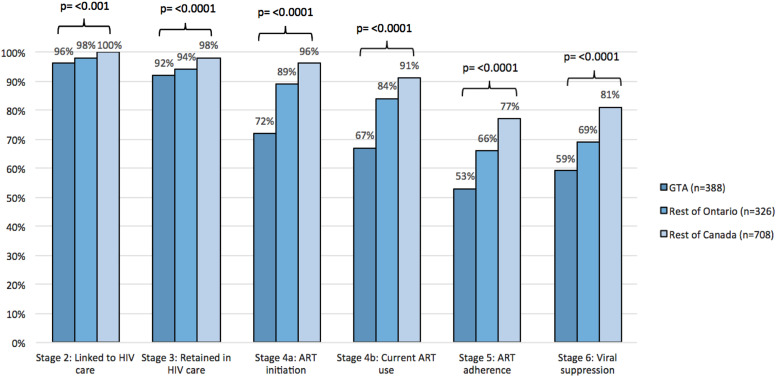
HIV cascade of care overall for women living with HIV in the GTA versus rest of Ontario versus in Quebec and British Columbia enrolled in CHIWOS^b^. ^b^Stage 1 (diagnosed with HIV) is not shown in the cascade because all women enrolled in CHIWOS were living with HIV (*N* = 1,424).

Of the 388 WLWH in the GTA enrolled in CHIWOS, 96% (*n* = 372/388) were linked to care (Figure 1). Of these, 92% (*n* = 357/388) were retained in care, 72% (*n* = 279/388) initiated ART, 69% (*n* = 268/388) were currently using ART; 53% (*n* = 206/388) were adherent, and 59% (*n* = 229/388) were virally suppressed. While stages linked to care and retained in care of the cascade were similar for women living in the GTA to those living in the rest of Ontario and in Quebec and BC, the stages of ART initiation, current ART use, ART adherence, and viral suppression were lower (*p*-value = <0.0001) (Figure 1).

**Figure 2.**
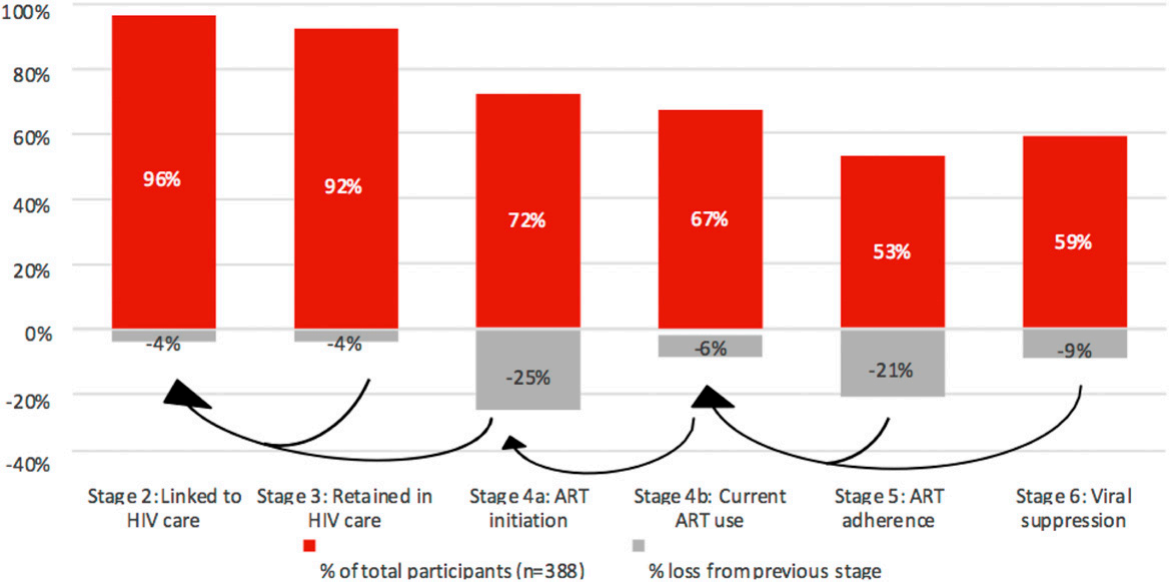
(a) HIV cascade of care for women living in the GTA (*n* = 388). (b) HIV cascade of care for women enrolled living in the rest of Ontario (*n* = 326). (c) HIV cascade of care for women enrolled living in Quebec and British Columbia (*n* = 708).

In terms of the 90-90-90 values, they were 100%-67%-88% for the GTA. For the rest of Ontario, they were 100%-84%-82%, and for Quebec and BC they were 100%-91%-89%. These analyses were done as a nested stage cascade system as per the 90-90-90 targets where the denominator for each step is the numerator of the prior stage. Attrition between stages is presented in Figure 2. The greatest attrition in the cascade for WLWH in the GTA occurred in ART initiation (−25%) and ART adherence (−21%) (Figure 2(a)). For WLWH in the rest of Ontario, and in Quebec and BC, the greatest attrition occurred in ART adherence followed by viral suppression (Figure 2(b) and (c)).

### Univariate and multivariable logistic regression for viral suppression for the entire cohort

Living in the GTA was associated with lower odds of achieving viral suppression compared to the rest of Ontario and to Quebec and BC in the univariate analyses. Women reporting older age, heterosexual identity, being separated/divorced/widowed (vs married/in relationship), not being a Canadian citizen, ACB identity (vs white), white (vs Indigenous) identity, higher education (high school or higher), stable housing, a personal annual income of ≥$20,000 CAD, food security, not experiencing violence as a child, a longer duration with HIV diagnosis, and higher resiliency scores had higher odds of viral suppression in unadjusted analyses (Table 2).

**Table 2.** Univariate and multivariable logistic regression analyses for viral suppression among women living with HIV enrolled in CHIWOS (*n* = 1,422).

Variables	Univariate analyses			Initial multivariable model (*n* = 1,013)			Final multivariable model (*n* = 1,218)		
	Odds Ratio	95% CI	*p*-value	Odds Ratio	95% CI	*p*-value	Odds Ratio	95% CI	*p*-value
Sociodemographic factor									
Location of living (*n* = 1,301)			<0.0001			<0.0001			<0.0001
GTA	ref			ref			ref		
Rest of Ontario	1.64	1.09, 2.46		2.15	1.25, 3.68		1.72	1.09, 2.72	
Quebec and British Columbia	2.17	1.54, 3.06		3.68	2.21, 6.16		2.42	1.62, 3.62	
Age (per year) (*n* = 1,301)	1.05	1.04, 1.07	<0.0001	1.05	1.03, 1.08	<0.0001	1.05	1.03, 1.06	<0.0001
Sexual orientation(*n* = 1,301)			<0.01			0.66			
Heterosexual	ref			ref					
LGBTTQ	0.52	0.35, 0.78		0.89	0.54, 1.48				
Legal relationship status (*n* = 1,300)			<0.001			0.95			
Married/common law/in relationship	ref			ref					
Single	0.97	0.70, 1.34		1.06	0.69, 1.63				
Separated/divorced/widowed/other	2.59	1.54, 4.36		1.00	0.51, 1.98				
Immigration status (*n* = 1,297)			<0.001			0.13			<0.0001
Canadian citizen	ref			ref			ref		
Landed immigrant/permanent resident	2.64	1.44, 4.86		1.70	0.70, 4.14		3.23	1.66, 6.26	
Refugee/protected person/other	2.96	1.28, 6.89		3.45	0.97, 12.34		4.77	1.96, 11.64	
Ethnicity (*n* = 1,298)			<0.0001			0.58			
Indigenous	0.57	0.40, 0.81		1.01	0.60, 1.72				
African/Caribbean/Black	2.35	1.53, 3.61		1.68	0.78, 3.63				
White	ref			ref					
Other ethnicity	0.92	0.49, 1.75		1.01	0.45, 2.27				
Education (*n* = 1,297)			0.01			0.09			0.01
Lower than high school	ref			ref			ref		
High school or higher	1.62	1.12, 2.34		1.57	0.93, 2.63		1.77	1.15, 2.73	
Housing status (*n* = 1,301)			<0.0001			0.02			0.01
Unstable	ref			ref			ref		
Stable	2.82	1.85, 4.30		1.93	1.14, 3.27		2.13	1.33, 3.39	
Personal annual income (CAD) (*n* = 1,274)			<0.01			0.13			0.05
<$20,000	ref			ref			ref		
≥$20,000	1.63	1.13, 2.36		1.49	0.89, 2.48		1.52	1.00, 2.31	
Food security (*n* = 1,296)			<0.001			0.51			
Food insecure	ref			ref					
Food secure	1.96	1.39, 2.75		1.17	0.73, 1.88				
History of violence as child (*n* = 1,193**)**			0.03			0.20			
Yes	ref			ref					
No	1.48	1.03, 2.13		1.36	0.85, 2.19				
Psychosocial factors										
Cannabis use (*n* = 1,281)			<0.01			0.06			
Yes (regularly/occasionally)	ref			ref					
Former	0.97	0.64, 1.46		0.52	0.31, 0.90				
Never	1.68	1.19, 2.39		0.71	0.43, 1.18				
History of incarceration (*n* = 1,301)			<0.001			0.12			
Never	3.19	1.87, 5.44		2.31	1.02, 5.22				
Ever, but not last year	1.93	1.11, 3.35		1.64	0.79, 3.40				
Last year	ref			ref					
Probable depression (CES-D) (per score increase) (*n* = 1,256)	1.01	0.98, 1.03	0.65	1.03	0.98, 1.07	0.22			
Clinical factors										
Duration of HIV diagnosis (year) (*n* = 1,260)			<0.0001			0.02			0.01
<6 years	ref			ref			ref		
6–14 years	2.04	1.42, 2.95		1.79	1.12, 2.84		1.75	1.16, 2.63	
>14 years	2.41	1.63, 3.57		1.90	1.13, 3.21		1.87	1.19, 2.96	
Hepatitis C (*n* = 1,296)			0.02			0.82			
Yes	ref			ref					
No	1.43	1.05, 1.96		1.06	0.63, 1.78				
Stigma and discrimination										
HIV-related stigma (HSS) (per score increase) (*n* = 1,284)	0.99	0.99, 1.00	0.09	1.00	0.99, 1.01	0.88			
Everyday discrimination racism (per score increase) (*n* = 1,276)	0.99	0.98, 1.00	0.10	0.99	0.96, 1.02	0.42			
Everyday discrimination sexism (per score increase) (*n* = 1,276)	0.99	0.97, 1.00	0.10	1.03	1.00, 1.06	0.10			
Well-being										
Resilience (per score increase) (*n* = 1,293)	1.03	1.01, 1.04	<0.01	1.03	1.00, 1.06	0.06	1.02	1.00, 1.04	0.02
Social support (MOS-SSS) (per score increase) (*n* = 1,254)	0.96	0.92, 0.99	0.02	0.99	0.94, 1.05	0.73			
Physical HRQOL (per score increase) (*n* = 1,286)	1.00	0.99, 1.01	0.85	1.00	0.99, 1.02	0.69			
Mental HRQOL (per score increase) (*n* = 1,286)	1.00	0.99, 1.01	0.84	1.01	0.99, 1.03	0.43			

Note: GTA: Greater Toronto Area; LGBTTQ: Lesbian, Gay, Bisexual, Transgender, Two-Spirit, and Queer or Questioning; HSS: HIV Stigma Scale; CES-D: Center for Epidemiological Studies Depression Scale; MOS-SSS: Social Support Scale; HRQOL: health-related quality of life.

We found lower odds of viral suppression for the GTA in comparison to the rest of Ontario and to Quebec and BC (Table 2) in the multivariable analyses. Viral suppression was also associated with higher age (aOR = 1.05, 95% CI: 1.03–1.06 per year), higher education (high school or higher) (aOR = 1.77, 95% CI: 1.15–2.73), housing stability (aOR = 2.13, 95% CI: 1.33–3.39), higher personal annual income (≥$20,000 CAD) (aOR = 1.52, 95% CI: 1.00–2.31), longer duration with HIV diagnosis (compared to <6 years) (aOR = 1.75, 95% CI: 1.16–2.63 for 6–14 years and aOR = 1.87, 95% CI: 1.19–2.96 for >14 years), and higher resiliency scores (aOR = 1.02, 95% CI: 1.00–1.04). The odds of virological suppression was higher in immigrants and refugees living with HIV compared to Canadian citizens (aOR = 3.23, 95% CI: 1.66–6.26 and aOR = 4.77, 95% CI: 1.96–11.64, respectively).

### Univariate and multivariable logistic regression for viral suppression for women living in the GTA

For WLWH in the GTA, viral suppression was associated with the same variables as those for the entire cohort in the univariate analyses, except that history of violence as a child, relationship status, history of incarceration, hepatitis c infection and resilience were no longer statistically significant, while depression was statistically significant (Table 3). In the final multivariable model, age (aOR = 1.10, 95% CI: 1.06–1.15), of ACB identity (compared to white) (aOR = 7.46, 95% CI: 3.02–18.43), having stable housing (aOR = 2.40, 95% CI: 1.07–5.37), having a longer duration of HIV diagnosis (compared to <6 years) (aOR = 2.90, 95% CI: 1.44–5.85 for 6–14 years and aOR = 2.86 95% CI: 1.20–6.95 for >14 years), and having higher physical health scores (aOR = 1.05, 95% CI: 1.02–1.08) were associated with increased odds of viral suppression for women living in the GTA.

**Table 3.** Univariate and multivariable logistic regression analyses for viral suppression among women living with HIV enrolled in CHIWOS who live in the Greater Toronto Area (GTA) (n=388).

Variables	Univariate analyses			Initial multivariable model (n=265)				Final multivariable model (n=313)			
	Odds Ratio	95% CI	*p* value	Odds Ratio		95% CI	*p* value	Odds Ratio	95% CI		*p* value
**Sociodemographic factors**											
**Age (per year) (n=334)**	**1.07**	**1.04, 1.11**	**<0.0001**	**1.12**		**1.05, 1.18**	**<0.001**	**1.10**	**1.06, 1.15**		**<0.0001**
**Sexual orientation (n=330)**			**0.02**				0.08				
Heterosexual	ref			ref							
LGBTTQ	**0.45**	**0.23, 0.86**		0.43		0.16, 1.12					
**Legal relationship status (n=333)**			0.26				0.05				
Married/common-law/in relationship	ref			ref							
Single	1.41	0.80, 2.48		1.95	0.85, 4.52						
Separated/divorced/widowed/other	2.06	0.81, 5.21		0.42	0.11, 1.67						
Immigration status* (n=333)			**<0.01**				0.55				
Canadian citizen	ref			ref							
Landed immigrant/permanent resident	**4.42**	**1.53, 12.76**		0.97	0.13, 7.48						
Refugee/protected person/ Other	**4.33**	**1.29, 14.56**		3.11	0.29, 33.43						
**Ethnicity (n=332)**			**<0.0001**				**0.04**			**<0.0001**	
White	ref			ref				ref			
Indigenous	**0.71**	**0.37, 1.34**		0.74	0.29, 1.90			0.85	0.40, 1.79		
African/Caribbean/Black	**6.21**	**2.81, 13.72**		**9.00**	**1.51, 53.79**			**7.46**	**3.02,** **18.43**		
Other ethnicity	1.07	0.44, 2.59		0.74	0.20, 2.70			0.82	0.29, 2.32		
**Education (n=333)**			0.60				0.65				
Lower than high school	ref			ref							
High school or higher	1.26	0.54, 2.95		0.69	0.14, 3.37						
**Housing status (n=334)**			**<0.01**				**0.01**			**0.03**	
Unstable	ref			ref				**ref**			
Stable	**2.83**	**1.45, 5.53**		**3.76**	**1.31, 10.76**			**2.40**	**1.07, 5.37**		
**Personal annual income CAD (n=324)**			0.12				0.08				
<$20,000	ref			ref							
=>$20,000 to $40,000	1.56	0.90, 2.70		0.42	0.16, 1.11						
**Food security (n=333)**			**0.02**				**0.04**				
Food insecure	ref			ref							
Food secure	**2.20**	**1.16, 4.15**		**2.78**	**1.04, 7.44**						
**History of violence as child (n=310)**			0.80				0.07				
Yes	ref			ref							
No	1.08	0.62, 1.88		2.19	0.94, 5.12						
**Psychosocial factors**											
**Cannabis use (n=325)**			**<0.01**				0.98				
Yes (regularly/occasionally)	ref			ref							
Former	1.13	0.45, 2.84		1.13	0.32, 3.95						
Never	**2.44**	**1.31, 4.51**		1.02	0.41, 2.55						
**History of incarceration (n=334)**			0.16				0.19				
Never	2.32	0.54, 10.02		7.32	0.78, 68.80						
Ever, but not last year	1.20	0.26, 5.56		8.36	0.85, 82.13						
Last year	ref			ref							
DK/PNTA	.			.							
**Probable depression (CES- D) (per score increase) (n=325)**	**1.05**	**1.01, 1.09**	**0.02**	0.92	0.85, 1.00		0.40				
**Clinical Factors**											
**Duration of HIV diagnosis (n=320)**			**<0.0001**				**0.01**			**<0.01**	
<6 years	ref			ref				ref			
6-14 years	**3.34**	**1.82, 6.14**		**3.22**	**1.37, 7.60**			**2.90**	**1.44, 5.85**		
>14 years	**3.15**	**1.56, 6.35**		**3.43**	**1.19, 9.90**			**2.86**	**1.20, 6.95**		
**Hepatitis C (n=334)**			0.15				0.68				
Yes	ref			ref							
No	1.72	0.82, 3.60		1.27	0.40, 3.98						
**Stigma & Discrimination**											
**HIV-related stigma (HSS) (per score increase)** **(n=331)**	1.01	0.99, 1.02	0.45	1.00	0.98, 1.03		0.71				
**Everyday discrimination racism (per score increase)** **(n=330)**	1.02	0.99, 1.04	0.25	**0.92**	**0.85, 1.00**		**0.04**				
**Everyday discrimination sexism (per score increase) (n=328)**	1.02	0.99, 1.04	0.25	**1.12**	**1.03, 1.22**		**<0.01**				
**Well-being**											
**Resilience (per unit score increase) (n=331)**	0.97	0.94, 1.01	0.17	1.04	0.97, 1.11		0.25				
**Social Support (MOS-SSS) (per score increase) (n=325)**	**0.88**	**0.83, 0.95**	**<0.001**	1.04	0.91, 1.18		0.56				
**Physical HRQOL (per score increase) (n=329)**	0.99	0.97, 1.01	0.34	1.04	0.99, 1.08		0.10	**1.05**	**1.02, 1.08**	**<0.01**	
**Mental HRQOL (per score increase) (n=329)**	**0.97**	**0.95, 0.99**	**<0.001**	**0.94**	**0.90, 1.00**		**0.03**				

Note: LGBTTQ, Lesbian, Gay, Bisexual, Transgender, Two-Spirit, and Queer or Questioning; DK/PTNAƚ, do not know/prefer not to answer; HSS, HIV Stigma Scale; CES-D, Center for Epidemiological Studies Depression Scale; MOS-SSS, Social Support Scale; HRQOL, health-related quality of life

## Discussion

We assessed sociodemographic and clinical differences as well as differences in the HIV care cascade stages between WLWH in the GTA, the largest city in Canada, versus the rest of Ontario and in Quebec and BC. Women living in the GTA were more likely to be younger, living with HIV for a shorter duration, not in a relationship, of ACB identity, gender and sexually diverse and born outside of Canada in comparison to WLWH in the rest of Ontario, Quebec and BC. Women living in the GTA had a higher education level and income, lower rates of food, and housing security compared to women living in the rest of Ontario, Quebec and BC. While women living in the GTA experienced more HIV-related stigma, racism, and sexism, they had lower depression scores, higher mental and physical health-related quality of life, higher resiliency scores, and higher social support scores than women in the rest of Ontario, Quebec and BC.

Although the UNAIDS 90-90-90 goals are nearly being met for WLWH in other parts of Canada, women living in the GTA are falling behind with surprising low rates of current ART use at 67% and viral suppression at 59%. We identified that younger age, being white (vs ACB identity), being more recently diagnosed with HIV, and having unstable housing were predictors of unsuppressed viremia – these key groups should be targeted with specific programming to improve engagement in care that could improve viral suppression rates. Overall, this information can inform endeavors such as the Toronto to Zero initiative, and to tailor programs for WLWH in the GTA to support ART retention and realizing viral suppression.

With such a high proportion of WLWH in the GTA identifying as ACB, social and health services specific for ACB women is essential. A great example of this is a dedicated community health center, Women’s Health in Women’s Hands.^28–30^ This community health center is a leader in Toronto and surrounding municipalities to improving health outcomes among Black and racialized women by promoting gender equality, economic opportunity, women-controlled prevention technologies. Appreciating the intersectional identities—and associated experiences of stigma and coping—among WLWH living in the GTA is essential to providing tailored social and health services alongside stigma reduction interventions in health care, education, employment, among other contexts.^31^ Furthermore, understanding the high rates of HIV-related stigma, racism, and sexism experienced by WLWH in the GTA supports the current movement of anti-oppression training, anti-Black and anti-Indigenous racism training, and cultural safety and humility training being conducted at healthcare and social services organizations across the GTA.

The low rates of current ART use (67%) and viral suppression (59%) amongst WLWH in the GTA is worrisome. Research by the Canadian HIV Observational Cohort Collaboration reported that 8358/9031 individuals (93%) engaged in care and achieved viral suppression which surpasses the UNAIDS 90-90-90 targets.^38^ It was reported that adults aged 29 or younger were less likely to experience viral suppression particularly among women with a history of IDU and a baseline CD4 cell count >200. Indigenous identity reported at ART initiation was also found to be strong predictors of viral suppression in this cohort. Findings from Benoit et al.^32^ not only echoed the same findings of poor viral suppression among PLWH in Canada, but also reported how younger adults who identify as Indigenous (54%) were less likely than non-Indigenous people (77%) to experience viral suppression. Indigenous women, who comprise 22% of CHIWOS participants, reported low rates of viral suppression in comparison to ACB and white women regardless of whether they lived in the GTA, in the rest of Ontario or in Quebec and BC. Our findings reinforce the need for a more equitable, diverse, and inclusive research funding system that places the voices of Indigenous and ACB people in the center of the research process.^33^ Further, to address the social and structural determinants of health, including pervasive anti-Indigenous racism, in addition to developing Indigenous-centred and culturally safe programs with and for younger Indigenous WLWH.

For many young WLWH, remaining in care can be difficult due to competing priorities (e.g. school, work, friends), co-morbidities (e.g. depressive symptoms), and trauma. Rapid ART start programs^34,35^ could have merit, especially for younger individuals with a more recent diagnosis. Rapid start programs have been developed with the idea of starting ART within 24 h of being diagnosed with HIV and have been associated with higher retention in care.^34,35^ In BC, the STOP HIV/AIDS Program has been rolled out with success, with BC now having higher rates of ART use and lower HIV incidence as compared to the rest of Canada.

We found that higher education level and income were associated with higher suppression rates in the GTA, rest of Ontario, Quebec and BC. Results presented in this study are similar to results from other studies where higher education and income levels were associated with higher rates of retention and viral suppression mainly due to increased access to HIV medical care.^36–39^ Martinez et al. reported that stigma undermines socio-economic status, and negatively impacts diagnosis and adherence to treatment.^40^ Their study followed 178 young adult females living with HIV who were enrolled in the Adolescent Trials Network and experiencing high levels or HIV stigma, and found that women were three times more likely to be non-adherent to their treatment compared to those with low HIV stigma concerns. Lipira et al. similarly found in the Unity Study, a multi-site study among African-American women with HIV in the United States, that greater levels of HIV-related stigma were less likely to be virally suppressed.^41^ Our findings were not consistent with the literature and calls for further insight into potential differences in the effects of HIV-related stigma’s contribution to diminished viral suppression across populations.

We also found that immigrant and refugee women had higher rates of viral suppression compared to women with Canadian citizenship. This is a testament to the Canadian immigration and refugee programs and public access to healthcare. Similar studies have found that recent immigrants to Canada were more likely to achieve viral suppression because they were more likely to be diagnosed at earlier stages of the disease than other persons, and that they perceived improved immune status as improving their immigration opportunities – this was especially true for women.^42,43^ Immigrants are likely highly motivated to engage in care, but further efforts are needed to improve immigrant women’s engagement in and adherence to HIV care and treatment.

An important finding is that housing stability was associated with viral suppression. This was the case for the rest of Ontario, and in Quebec and BC, but seemed to be more important in the GTA given the housing crisis and unaffordability of housing in Toronto and its surrounding municipalities. The importance of housing stability is highlighted in several other studies. Riley et al.^44^ reported that issues of poverty and homelessness were a major barrier to achieving viral suppression for WLWH. Their study, the San Francisco-based Shelter, Health and Drug Outcomes among Women Study, followed 120 WLWH who experience homelessness and found that 60% had ≥1 unsuppressed viral load over the 3 year follow up and were 11% more likely to have detectable viremia for every 10 nights spent sleeping on the street, and 16% more likely to have detectable viremia for every 10 nights spent sleeping in a shelter. Results presented here are similar to those by Rajabiun et al.^45^ of PLWH enrolled in the Building a Medical Home for Multiply Diagnosed HIV-positive Homeless Populations initiative. Access to and uptake of housing programs by PLWH was associated with increased viral suppression over a 12-month period. Active substance use was not a predictor of housing instability in either study. Our findings reinforce this literature showing that targeting stable housing supports is essential for HIV care.

The study was not without its limitations. The use of self-reported measures, including resilience, HIV-related stigma, depression, and violence, may have been impacted by considerations of social desirability, while others may be affected by recall bias causing an overestimation of outcomes in the study. Similarly, self-reported viral response risk biases; however, a prior study shows the validity of self-reported measures of viral load among women with HIV in BC enrolled in CHIWOS.^27^ There may also be sampling biases between the regions. Despite these limitations, the findings from this study extend our understanding of viral suppression and its association to the health outcomes of WLWH and suggests a number of additional areas for future research and intervention efforts.

In conclusion, WLWH in the GTA were found to be sociodemographically and clinically distinct and less likely to be currently taking ART (67%) and have viral suppression (59%) compared to those living in the rest of Ontario (84% and 69%) and in Quebec and BC (91% and 81%). The GTA is falling short of reaching the Fast Track City 90-90-90 targets for WLWH, and should consider adopting more intense outreach programming such as BC’s STOP HIV/AIDS program and tailored programming targeting younger women, and women more recently diagnosed with HIV. The GTA has its work cut out to meet its targets and should look to other Fast Track cities efforts, including those from Amsterdam and Paris who have successfully met the 90-90-90 goal, to reach the last 90% benchmark for viral suppression. Qualitative studies are needed to identify factors strongly associated with HIV virological failure among WLWH and inform policies to close the gaps created by inequalities pointed out by this paper.
